# The role of the sirtuin family in cartilage and osteoarthritis: molecular mechanisms and therapeutic targets

**DOI:** 10.1186/s13075-022-02983-8

**Published:** 2022-12-31

**Authors:** Kaibo Sun, Yuangang Wu, Yi Zeng, Jiawen Xu, Limin Wu, Mingyang Li, Bin Shen

**Affiliations:** Department of Orthopedics Surgery, Orthopedic Research Institute, West China Hospital, Sichuan University, Chengdu, 610041 Sichuan China

**Keywords:** Osteoarthritis, Sirtuin, Cartilage destruction, Chondrocyte, Mitochondrial dysfunction

## Abstract

Osteoarthritis (OA) is mainly characterized by the progressive destruction of articular cartilage. Mounting studies have revealed that disruption of extracellular matrix (ECM) homeostasis, aberrant chondrocyte metabolism, an increase in the number of senescent chondrocytes and abnormal activation of cell death such as chondrocyte apoptosis and autophagy, are the crucial steps in OA development. Additionally, mitochondrial dysfunction also participates in the abovementioned processes and is the key element of OA pathogenesis. Sirtuin (SIRT) is a family of nicotinamide adenine dinucleotide (NAD^+^)-dependent protein deacetylases that can actively participate and primarily regulate chondrocyte function in OA pathophysiological processes. Some members of the SIRT family located in mitochondria can regulate mitochondrial function and mediate mitochondrial homeostasis via deacetylation to protect chondrocytes. In addition, SIRT can maintain ECM homeostasis, regulate chondrocyte metabolism, inhibit chondrocyte apoptosis and autophagy, and prevent chondrocyte senescence in cartilage by exerting its deacetylation activity. However, the molecular mechanism of the SIRT family against the onset and development of OA remains poorly elucidated. In this review, we will discuss the potential protective role of SIRT in the progression of OA and summarize several sirtuin-activating molecules as well as their potential therapeutic applications for OA.

## Introduction

Osteoarthritis (OA) is the most common chronic and degenerative joint disease that affects elderly people, causing debilitating pain, mobility loss, and possibly disability, as well as a significant socioeconomic burden [1]. Cartilage degeneration has been identified as a central feature in the progression of OA. Chondrocytes are surrounded by an abundant extracellular matrix (ECM), and as the only cell type existing in cartilage, they are mainly responsible for the maintenance of the surrounding microenvironment and cartilage homeostasis.

Multiple lines of evidence indicate that chondrocyte dysfunction appears to be a crucial factor in the progression of OA [2]. In the early stage of OA, chondrocytes experience a pathological shift, increasing the production of matrix-remodelling enzymes. The synthetic capacity of chondrocytes is overwhelmed by processes that promote matrix breakdown, contributing to cartilage degradation. Moreover, the progression of OA is also attributed to abnormal chondrocyte metabolism, and emerging evidence has revealed that OA and other metabolic diseases have common pathways, including lipid metabolism [3, 4]. Recent studies have demonstrated that abnormal lipid accumulation is connected with cartilage degradation [5]. In addition, chondrocyte death is an important contributor to OA development. The senescence of chondrocytes also increases with age, resulting in diminished cellular proliferation and tissue regeneration.

Unlike many other well-vascularized tissues with higher levels of oxygenation, chondrocytes surround the lower oxygen environment. The evidence indicated that reactive oxygen species (ROS), which are a major cause of oxidative stress, were overproduced in chondrocytes in end-stage OA cartilage [6]. Mitochondrial quality control could participate in the clearance of ROS and prevent mitochondrial dysfunction through mitochondrial biogenesis, mitophagy, dynamics, mitochondrial redox and the newly discovered mitocytosis [7–9]. Mitochondrial dysfunction also causes chondrocyte apoptosis and autophagy, both of which contribute to cartilage degradation [4, 10, 11]. Therefore, mitochondrial function plays an important role in maintaining the homeostasis of chondrocytes.

Sirtuin (SIRT) is a group of nicotinamide adenine dinucleotide-dependent histone deacetylases that can deacetylate certain proteins and play a key role in the regulation of cell physiological and pathological processes. Of the seven SIRTs, mammalian class III histone deacetylases, sirtuin 1 (SIRT1) is localized in the nucleus and cytoplasm, sirtuin 2 (SIRT2) is present in the cytoplasm; sirtuin 3 (SIRT3), sirtuin 4 (SIRT4), and sirtuin 5 (SIRT5) have a primary mitochondrial localization; and sirtuin 6 (SIRT6) and sirtuin 7 (SIRT7) are primarily found in the nucleus. In addition to deacetylase activity, the SIRT family has many functions such as demalonylase, desuccinylase, deglutarylase activity and ADP-ribosylation. For example, SIRT3-5 can lead to ADP-ribosylation, SIRT5 has strong demalonylase and desuccinylase activity, and SIRT7 plays a role in the deglutarylase activity [12–14].

Extensive studies have revealed that some sirtuin-activating molecules have beneficial effects on encouraging chondrocyte survival in cartilage and show promising early results for treating OA [15]. However, evidence of the benefit is currently limited by a lack of evidence testing the specificity and efficacy of these drugs. This review aims to explore the molecular mechanisms related to SIRT during the progression of OA and summarize potential therapeutic strategies targeting SIRT directly.

**Fig. 1:**
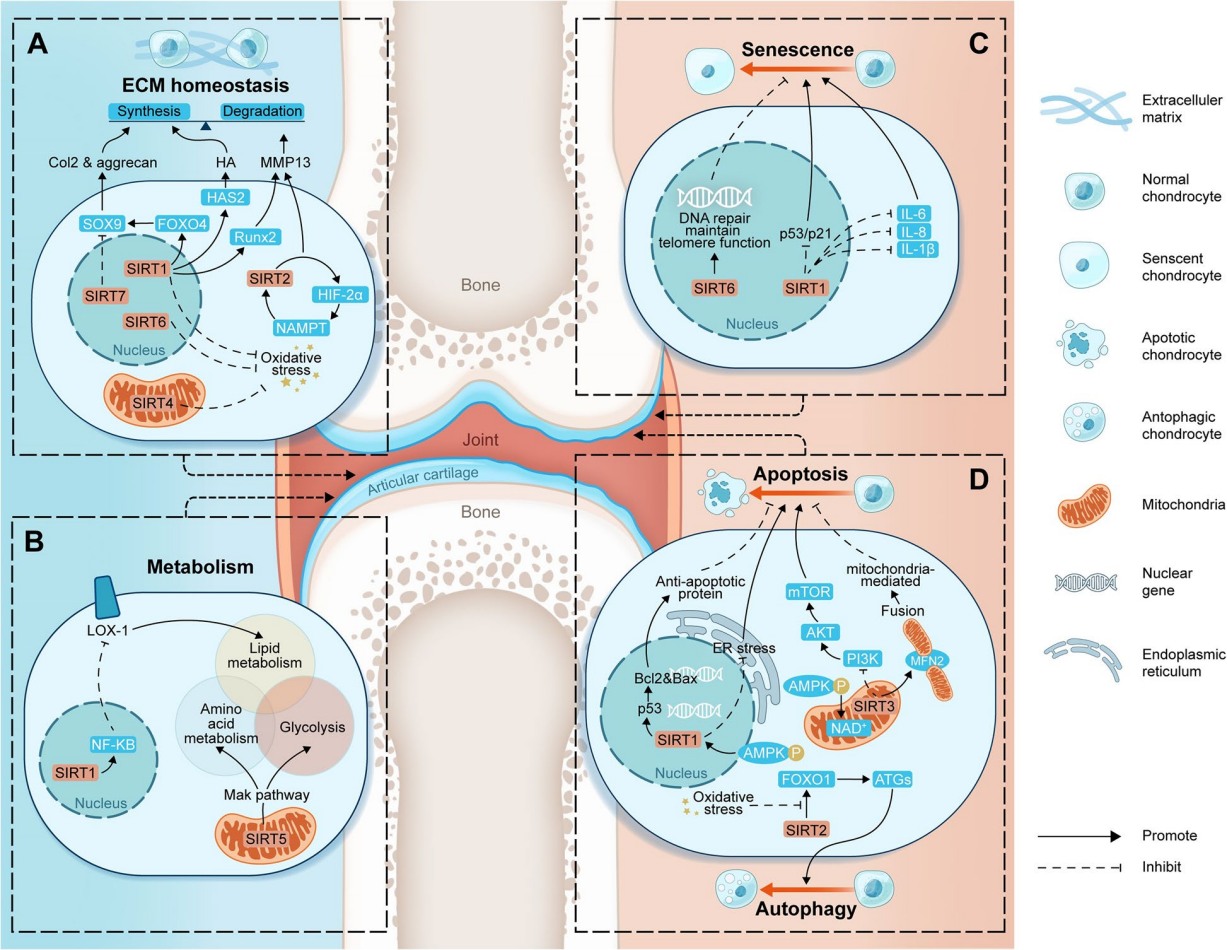
The role of the SIRT family in maintaining chondrocyte function. **A** SIRT in maintaining ECM homeostasis. **B** SIRT in regulating chondrocyte metabolism. **C** SIRT in preventing chondrocyte senescence. **D** SIRT in decreasing chondrocyte apoptosis and enhancing chondrocyte autophagy. Abbreviations: SIRT, sirtuins; SIRT1, sirtuin 1; SIRT2, sirtuin 2; SIRT3, sirtuin 3; SIRT4, sirtuin 4; SIRT5, sirtuin 5; SIRT6, sirtuin 6; SIRT7, sirtuin 7; ECM, extracellular matrix; Col2, type 2 collagen; HA, hyaluronan; MMP-13, matrix metalloproteinase-13; SOX9, SRY-Box transcription factor 9; FOXO4, forkhead box O-4; HAS2, hyaluronan synthase 2; Runx2, Runt-Related Transcription Factor 2; NAMPT, nicotinamide phosphoribosyltransferase; HIF-2α, hypoxia-inducible factor-2α; LOX-1, lectin-like oxidized low-density lipoprotein receptor-1; NF-κB, nuclear factor-κB; Mak, protein lysine malonylation; IL-1β, interleukin-1β; IL-6, interleukin-6; IL-8, interleukin-8; Bcl-2, B-cell lymphoma-2; Bax, Bcl-2-associated X; ER, endoplasmic reticulum; mTOR, mammalian target of rapamycin; PI3K, phosphoinositide 3-kinase; Akt, protein kinase B; NAD + , nicotinamide adenine dinucleotide; p-AMPK, phospho-adenosine monophosphate-activated protein kinase; MFN2, mitofusin-2; FOXO1, forkhead box O-1; ATGs, autophagy-related proteins

**Fig. 2:**
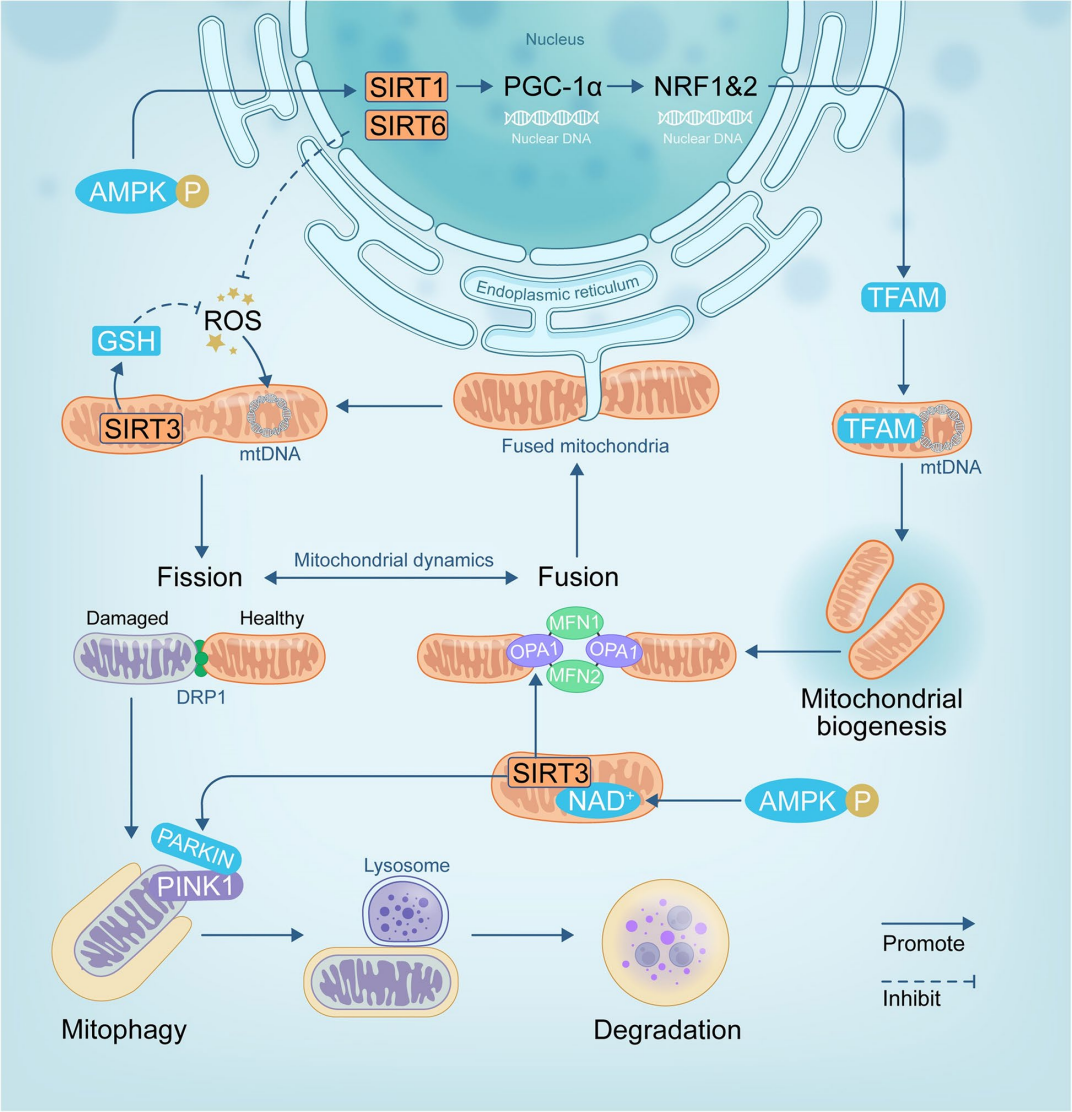
The role of the SIRT family in mediating mitochondrial quality control in chondrocytes. SIRT1 can deacetylate PGC-1α, and then coactivate the transcription of NRF-1 or NRF-2, enhancing TFAM expression, which this process promotes mitochondrial biogenesis. SIRT3 could deacetylate OPA1 to promote mitochondrial dynamics, deacetylate Parkin to contribute to mitophagy, and elevate the level of GSH to reduce ROS to maintain mitochondrial homeostasis in chondrocytes. Abbreviations: SIRT, sirtuins; SIRT1, sirtuin 1; SIRT3, sirtuin 3; SIRT6, sirtuin 6; p-AMPK, phospho-adenosine monophosphate-activated protein kinase; PGC-1α, peroxisome proliferator-activated receptor Coactivator-1α; NRF-1, nuclear respiratory factor 1; NRF-2, nuclear respiratory factor 2; TFAM, mitochondrial transcription factor A; NAD^+^, nicotinamide adenine dinucleotide; OPA1, optic atrophy-1; MFN1, mitofusin-1; MFN2, mitofusin-2; DRP1, dynamin-related protein 1; PINK1:, PTEN-induced putative kinase-1; GSH, glutathione; ROS, reactive oxygen species

Accumulating evidence has recently documented that the functional impairment of chondrocytes, which hardly maintain homeostasis in response to changing stress conditions, is a crucial factor in the pathophysiology of OA [2]. The SIRT family was reported to maintain chondrocyte function by maintaining ECM homeostasis, regulating chondrocyte metabolism, preventing chondrocyte senescence, decreasing chondrocyte apoptosis and enhancing chondrocyte autophagy (Fig. 1). Mounting evidence suggests that mitochondrial dysfunction may play a role in the onset of ageing and degenerative diseases such as OA [4, 10, 16, 17]. SIRT plays a regulatory role in mediating chondrocyte mitochondrial quality control and protecting against the progression of OA (Fig. 2).

### The role of SIRT1, SIRT2, SIRT4, SIRT6 and SIRT7 in maintaining ECM homeostasis in OA

Disruption of ECM homeostasis is one of the key elements of OA progression. Sirtuin 1 (SIRT1) is critical in maintaining cartilage health by promoting ECM homeostasis in the regulation of the expression of ECM-related proteins. The inhibition of SIRT1 may reduce the expression of hyaluronan synthase 2 (HAS2), which, in turn, downregulates hyaluronan (HA) production [18, 19]. The reduction in HA contributes to the progression of OA. SIRT1 modulates matrix metalloproteinase 13 (MMP-13) production from chondrocytes in OA by regulating the expression of Runt-Related Transcription Factor 2 (Runx2), a promotor of MMP-13 expression [20]. On the other hand, Oh, H. et al. demonstrated that the reciprocal control of the hypoxia-inducible factor-2α (HIF-2α) and nicotinamide phosphoribosyltransferase (NAMPT)-SIRT2 pathways are required for the development of catabolic matrix metalloproteinases such as MMP-13 [21]. Moreover, SIRT1 can deacetylate forkhead box O-4 (FOXO4) and then activate SRY-Box transcription factor 9 (SOX9), which can control the expression of cartilage-specific genes, allowing cartilage to maintain its ECM homeostasis [22]. In contrast, SIRT7 appears to inhibit the transcriptional activity of SOX9. Korogi, W et. al. demonstrated that inhibition of SIRT7 increased the mRNA expression of ECM components such as collagen II and aggrecan in chondrocytes [23].

Oxidative stress has been proposed as a driver of the catabolic and anabolic signalling imbalance in cartilage that results in progressive matrix degradation. Dai, Y. et al. revealed that silencing SIRT4 induced healthy chondrocytes to downregulate aggrecan, collagen II and antioxidant enzymes, while overexpression of SIRT4 suppressed the inflammatory response and reduced oxidative stress [24]. Collins, J. An et al. demonstrated that overexpression of SIRT6 decreased catabolic signal pathways such as oxidative stress-induced accumulation of nuclear phosphorylated p65 [25].

### The role of SIRT1 and SIRT5 in regulating chondrocyte metabolism in OA

The metabolic alteration of chondrocytes was reported to be another pathological factor in OA progression. When exposed to environmental stress, chondrocytes tend to change metabolic pathways involved in enhanced anaerobic glycolysis and alter lipid and amino acid metabolism [2]. At the molecular level, metabolic dysregulation in OA was linked to decreased SIRT activity. For instance, SIRT1 enhances catabolic activity and fatty acid oxidation. Papageorgiou et al. revealed that SIRT1 activated by resveratrol regulated lipid homeostasis in late-stage OA by decreasing the expression of lectin-like oxidized low-density lipoprotein receptor-1 (LOX-1) via nuclear factor-κB (NF-κB) deacetylation [26]. SIRT5 has been also proposed to regulate chondrocyte metabolism, including amino acid metabolism, the tricarboxylic acid cycle (TCA) cycle and glycolysis, and the SIRT5-mediated protein lysine malonylation (Mak) pathway was proven to regulate chondrocyte cellular metabolism in obesity-associated OA development [27]. The deletion of SIRT5 leads to an increase in the level of posttranslationally modified hundreds of metabolic proteins in cartilage, and it is possible to promote the early OA-like phenotype [28].

### The role of SIRT1 and SIRT6 in preventing chondrocyte senescence in OA

Chondrocyte senescence is thought to play a crucial role in the onset of cartilage breakdown and the progression of OA. Senescence is a process in which damaged cells enter permanent cell cycle arrest and avoid apoptosis while also transforming their secretory activity into the senescence-associated secretory phenotype (SASP) [29]. The quantity of senescent chondrocytes grows with age and leads to osteoarthritis; however, removing senescent cells from articular cartilage can help prevent OA. Recent studies have shown that SIRT exerts a beneficial effect to protect chondrocytes from senescence by increasing cartilage-specific gene expression and inhibiting apoptosis.

SIRT1 has been revealed to play a role in preventing the onset of chondrocyte senescence. For example, SIRT1 suppresses the expression of SASP factors such as interleukin-6 (IL-6), interleukin-8 (IL-8) and interleukin-1β (IL-1β) [29]. Loss of SIRT1 in cartilage causes the acceleration of OA progression by aberrantly activating the p53/p21-mediated SASP [30].

SIRT6 suppression resulted in an accumulation of DNA damage and telomere dysfunction, eventually leading to cellular senescence, strongly suggesting that SIRT6 is involved in chondrocyte senescence regulation. Nagai, K et al. revealed that SIRT6 prevented premature senescence in human chondrocytes by coordinating DNA repair and preserving appropriate telomere function [31]. Several studies have also supported the idea that SIRT6 promotes DNA repair under stress and protects against telomere dysfunction through deacetylation [32]. Zhao et al. showed that Sirt6 inhibited the acetylation of p27, which is highly acetylated with a prolonged half-life during cellular senescence, leading to its degradation via the ubiquitin–proteasome pathway and postponing cellular senescence [33]. Moreover, Wu, Y et al. found that the level of SIRT6 in chondrocytes of OA patients was much lower than that in normal individuals and that overexpression of SIRT6 can slow the progression of OA by reducing chondrocyte senescence [34].

### The role of SIRT1, SIRT2 and SIRT3 in decreasing chondrocyte apoptosis and enhancing autophagy in OA

A growing body of evidence implies that chondrocyte apoptosis plays a role in articular cartilage degradation. With OA development, the synthetic capabilities of articular chondrocytes are gradually overwhelmed by excessive chondrocyte apoptosis.

SIRT1 regulates apoptosis in human chondrocytes by modulating mitochondria-related apoptotic signals through B-cell lymphoma-2 (Bcl-2) and Bcl-2-associated X (Bax) translocation [35]. When the expression of SIRT1 is suppressed, the acetylation of p53 is increased, leading to dysregulation of the downstream genes Bax and Bcl-2 [36]. The aberrant activation of p53 induced by acetylation-mediated senescence is associated with a secretory phenotype, hypertrophy and apoptosis, accelerating the progression of OA [30]. When SIRT1 was increased by resveratrol, p53 was decreased, leading to alleviation of OA progression [37].

Endoplasmic reticulum (ER) stress is one of the mechanisms that mediate chondrocyte apoptosis, and it severely affects the secretory activity of chondrocytes, resulting in disordered cartilage homeostasis [38]. One of the major functions of activating SIRT is targeting ER stress, thereby reducing the death of chondrocytes. Activating the SIRT1/adenosine monophosphate-activated protein kinase (AMPK) signalling pathway may reduce chondrocyte apoptosis in the knee joints by inhibiting ER stress. Furthermore, Liu, M et al. demonstrated that mitofusin-2 (MFN2) overexpression, which was activated via the AMPK/SIRT3 signalling pathway, improved mitochondrial fusion in cells, followed by improved mitophagy and increased mitochondrial membrane potential, resulting in the prevention of mitochondria-mediated apoptosis [39]. In addition, Xu, K et al. stated that the overexpression of SIRT3 reduced chondrocyte apoptosis by blocking the phosphatidylinositol-3-kinase (PI3K)/protein kinase B (PKB/AKT)/mammalian target of rapamycin (mTOR) signalling pathway and then attenuated the extent of chondrocyte degeneration [40].

There is significant evidence suggesting that enhancing autophagy in chondrocytes can help slow the progression of OA. As the autophagy initiator primarily in the nucleus, SIRT1 can regulate the binding of transcription factors to promoter regions of the autophagy gene [41]. In addition, SIRT2 can promote the activation of autophagy by deacetylating forkhead box O-1 (FOXO1), which regulates the fusion of autophagosomes and lysosomes by interacting directly with autophagy-related proteins (ATGs) [42]. Once stress stimulates the separation of SIRT1 and SIRT2 from their substrate FOXO1, the acetylation of FOXO1 considerably increases, resulting in decreased autophagy.

### The role of SIRT1, SIRT3 and SIRT6 in regulating mitochondrial dysfunction in OA

#### The role of SIRT1 and SIRT3 in promoting mitochondrial biogenesis in OA

In human OA chondrocytes, mitochondrial biogenesis is impaired which promotes chondrocyte procatabolic responses. Activation of the SIRT1- peroxisome proliferator-activated receptor-γ coactivator-1α (PGC-1α) pathway increases mitochondrial biogenesis in chondrocytes, thus slowing the progression of OA. The SIRT1-PGC1α pathway can lead to the stimulation of mitochondrial gene expression [43]. SIRT1 deficiency inhibits mitochondrial biogenesis, resulting in a decrease in mitochondrial function [44]. In general, the localization of PGC-1α in the nucleus was transformed when acetylated, and thus transcriptional activity was inhibited. In chondrocytes, activation of AMPK increases nuclear PGC-1α/nuclear respiratory factor 1 (NRF-1) /nuclear respiratory factor 2 (NRF-2) expression and promotes mitochondrial transcription factor A (TFAM) transport, which promotes the replication of mitochondrial DNA (mtDNA) and gene expression [45].

Additionally, overexpression of SIRT-3 stimulated mitochondrial biogenesis. In human chondrocytes, after being activated by AMPK, SIRT-3 reduced mtDNA4977 deletion and maintained mtDNA integrity [15]. When SIRT3 was silenced using siRNA in rat cardiomyoblasts (H9C2 cells), the acetylation status of TFAM was increased, corresponding to a decrease in mtDNA binding activity of TFAM and reducing transcription of mitochondrial DNA-encoded genes [46]. Further research is needed to verify whether SIRT3 directly targets TFAM in chondrocytes.

#### The role of SIRT1 and SIRT3 in regulating mitochondrial dynamics in OA

Mitochondrial dynamics is a network of dynamic processes to adapt to the cell cycle, involving mitochondrial fusion and fission. Fusion creates a more linked mitochondrial network that improves communication with the ER, while fission produces smaller and more distinct mitochondria that generate more ROS and promote mitophagy, two processes that are both mediated by guanosine triphosphatase (GTPase).

Mitochondrial fusion allows matrix content to permeate throughout mitochondria, reducing the number of mitochondrial DNA mutations and oxidized proteins. This process is facilitated by optic atrophy-1 (OPA1) in the inner mitochondrial membrane, as well as mitofusin-1 (MFN1) and MFN2 in the outer mitochondrial membrane [47]. Enhancing the expression of OPA1 can promote mitochondrial fusion in chondrocytes [48]. In addition, Xu, L et al. indicated that elevated MFN2 contributes to metabolic changes and inflammation in rat chondrocytes, whereas decreased MFN2 reduces inflammation and slows OA progression [49]. SIRT3 contributes to promoting mitochondrial function by regulating mitochondrial dynamics by deacetylating OPA1 and elevating its GTPase activity [7]. In human articular chondrocytes, SIRT3 regulates mitochondrial dynamics by deacetylating and activating OPA1 to reduce chondrocyte apoptosis [50].

Mitochondrial fission is mediated by dynamin-related protein 1 (DRP1), which translocates to the outer mitochondrial membrane and forms a ring-like structure once activated to constrict and divide mitochondria [51]. Under disease conditions, the process of mitochondrial fission enhances the removal of damaged mitochondria, which promotes apoptotic programs [17]. Ding, M et al. indicated that the expression of SIRT1 negatively regulates DRP1-mediated mitochondrial fission directly via the SIRT1/PGC-1α-dependent pathway in H9C2 cells [52]. However, there have been no studies on chondrocytes, and it will be of great interest to explore the distinct functional roles of SIRT in mitochondrial fission in chondrocytes.

#### The role of SIRT3 in contributing to mitophagy in OA

Mitophagy may protect chondrocytes from oxidative stress by removing damaged and dysfunctional mitochondria. Mitophagy impairment causes the progressive accumulation of defective mitochondria, resulting in cartilage degeneration and OA. Parkin-mediated mitophagy is a key mechanism for clearing damaged mitochondria and improving OA chondrocyte survival [53]. PTEN-induced putative kinase-1 (PINK1), which is linked to the functional and health statuses of mitochondria, usually accumulates on the outer membrane of damaged mitochondria to recruit and activate Parkin. Parkin ubiquitinates and specifically incorporates a wide range of outer mitochondrial membrane proteins into mitophagy.

The SIRT family has recently been discovered to play a significant role in the removal of damaged mitochondria via mitophagy [43]. SIRT3 activated by metformin can reduce oxidative stress and rectify the imbalance of anabolism and catabolism induced by IL-1β via the PINK-1/Parkin signalling pathway in chondrocytes [54]. Polletta, L et al. observed that mitophagy increased in SIRT5-silenced cells and found that SIRT5 also controls mitophagy by controlling glutamine metabolism [55]. This result suggested that SIRT5 might also be a potential regulator of mitophagy, but this hypothesis needs further confirmation in OA.

#### The role of SIRT3 and SIRT6 in maintaining mitochondrial redox homeostasis in OA

Mitochondrial dysfunction increases the accumulation of ROS. Excessive generation of ROS can compromise the integrity and repair capacity of mtDNA, leading to an increase in mitochondrial ROS levels and increased oxidative damage. ROS were overproduced in OA cartilage due to oxidative stress more than in healthy cartilage. However, some SIRTs can maintain mitochondrial redox homeostasis and protect against mitochondrial oxidative damage by regulating the deacetylation of downstream related proteins, playing a pivotal role in mitochondrial function.

SIRT3 was proven to be a crucial regulator of promoting resistance to oxidative stress and maintaining glutathione (GSH) redox homeostasis in chondrocytes [56]. Zhu, S et al. found that SIRT3 deficiency delayed the rate of increasing glutathione content and GSH:oxidized glutathione (GSSG) ratio under oxidative stress in OA-related models [56]. Collins, J. An et al. demonstrated that enhancement of SIRT6 contributed to chondrocyte redox homeostasis by regulating antioxidant proteins of the peroxiredoxin catalytic cycle [25].

## Potential therapeutic strategies for OA-targeted SIRT

Currently, some exosome cargos, such as noncoding RNAs (ncRNAs) and proteins, have been reported to deliver multiple bioactive molecules to regulate cartilage behaviour, and are promising candidates for OA treatment [57]. Recent findings suggested that targeting SIRT may be a promising approach for OA therapy (Table 1).

**Table 1:** The mechanisms of the sirtuin-activating molecules delay the progression of OA

Sirtuin-activating molecules	Target or pathway	Mechanisms	Reference
Curcumin Quercetin	SIRT1	Inhibit chondrocyte apoptosis by reducing the ER stress	[58]
	SIRT1/AMPK	Attenuate oxidative stress-induced apoptosis by reducing the ER stress	[59]
Safflower yellow	NF-κB/SIRT1/AMPK	Inhibit inflammation by reducing the ER stress	[60]
Silymarin	SIRT1	Improve ECM homeostasis via deacetylating SOX9	[61]
Cyanidin	SIRT6/NF-κB	Delay ECM degradation by inhibiting the inflammatory response	[62]
Irisin	SIRT3	Retain mitochondrial biogenesis and attenuate mitophagy	[50]
Dihydromyricetin	SIRT3	Maintain mitochondrial homeostasis	[63]
Metformin	SIRT3	Enhance PINK1/Parkin-dependent mitophagy	[54]
Hydroxytyrosol	SIRT1	Protect chondrocytes by regulating the level of microRNA-9	[64]
	SIRT6	Mediate autophagy by reducing the inflammatory response	[65]

*Abbreviations*: *OA* osteoarthritis, *SIRT1* sirtuin 1, *SIRT3*sirtuin 3, *SIRT6* sirtuin 6, *AMPK* Adenosine monophosphate-activated protein kinase, ER Endoplasmic reticulum, *PINK-1* PTEN-induced putative kinase-1, *NF-κB* nuclear factor-κB, *ECM* Extracellular matrix, *SOX9* SRY-Box transcription factor 9

Inhibiting ER stress-induced chondrocyte apoptosis by activating SIRT1 may reduce cartilage degradation in OA [38]. In a rat OA model, Feng, K et al. discovered that quercetin and curcumin may reduce articular cartilage degradation and block chondrocyte apoptosis by reducing the ER stress response through SIRT1 activation [58, 59]. Additionally, safflower yellow can attenuate ER stress by activating the SIRT1/AMPK signalling pathway, thereby protecting chondrocytes and inhibiting inflammation, which suggests that targeting SIRT1 appears to be a viable treatment for OA [60].

In addition, SIRT1 also positively affects the ability of chondrocytes to synthesize ECM [18]. Wu, W. T et al. found that silymarin could improve ECM homeostasis through activation of SIRT1; thus, it may be a potential therapy for early-stage knee OA [61]. In addition, Jiang, C et al. showed that cyanidin may be able to delay the degradation of ECM by modulating the SIRT6/NF-κB signalling axis and ameliorating the progression of OA. [62].

Accumulating evidence has recently shown that cyanidin regulates a variety of mitochondrial proteins in chondrocytes to maintain mitochondrial homeostasis [7]. Wang, F. S et al. revealed that irisin retained mitochondrial biogenesis and attenuated mitophagy in IL-1β-induced inflamed chondrocytes by promoting SIRT3 signalling [50]. Wang, J et al. showed that dihydromyricetin protects rat chondrocytes from TNF-induced cartilage degradation by preserving mitochondrial homeostasis and activating SIRT3 [63]. Furthermore, Wang, C et al. also suggested that metformin could enhance SIRT3-mediated PINK1/Parkin-dependent mitophagy in chondrocytes, leading to suppression of IL-1β-induced OA-like inflammatory changes [54].

Inhibition of oxidative stress and the inflammatory response is a promising approach for OA prevention. In response to oxidative stress conditions, D'Adamo, S et al. implicated that hydroxytyrosol (HT) could regulate the level of microRNA-9 by targeting SIRT1, leading to the protective action of chondrocytes [64]. Furthermore, Zhi, L. Q et al. proposed that HT may reduce chondrocyte inflammatory responses via SIRT6-mediated autophagy [65].

## Conclusion

With the precise mechanisms partly elucidated in recent studies, SIRT may represent good candidates for the treatment of OA. SIRT is essential for maintaining cartilage homeostasis by regulating ECM homeostasis and chondrocyte metabolism, preventing chondrocyte senescence, decreasing chondrocyte apoptosis and enhancing chondrocyte autophagy. In addition, SIRT can deacetylate a range of targets in the regulation of mitochondrial function in mitochondrial biogenesis, mitophagy, mitochondrial dynamics and antioxidant pathways. Furthermore, recent studies have revealed that many sirtuin-activating molecules may have beneficial effects on chondrocyte survival and cartilage anabolism. Several small-molecule effectors targeting SIRT are promising in clinical trials and preclinical assessments of OA. However, the progression of treatment for OA from the laboratory to the clinic needs additional investigation, which should specifically focus on SIRT and the related molecular pathways in the future.

## Data Availability

Not applicable.

## Abbreviations

OA: Osteoarthritis
ECM: Extracellular matrix
ROS: Reactive oxygen species
SIRT: Sirtuins
SIRT1: Sirtuin 1
SIRT2: Sirtuin 2
SIRT3: Sirtuin 3
SIRT4: Sirtuin 4
SIRT5: Sirtuin 5
SIRT6: Sirtuin 6
SIRT7: Sirtuin 7
HAS2: Hyaluronan synthase 2
HA: Hyaluronan
MMP-13: Matrix metalloproteinase 13
Runx2: Runt-Related Transcription Factor 2
HIF-2α: Hypoxia-inducible factor-2α
NAMPT: Nicotinamide phosphoribosyltransferase
FOXO4: Forkhead box O-4
SOX9: SRY-Box transcription factor 9
LOX-1: Lectin-like oxidized low-density lipoprotein receptor-1
NF-κB: Nuclear factor-κB
TCA: Tricarboxylic acid cycle
Mak: Protein lysine malonylation
SASP: Senescence-associated secretory phenotype
IL-6: Interleukin-6
IL-8: Interleukin-8
IL-1β: Interleukin-1β
Bcl-2: Genes B-cell lymphoma-2
Bax: Bcl-2-associated X
ER: Endoplasmic reticulum
AMPK: Adenosine monophosphate-activated protein kinase
MFN2: Mitofusin-2
PI3K: Phosphatidylinositol-3-kinase
PKB/AKT: Protein kinase B
mTOR: Mammalian target of rapamycin
FOXO1: Forkhead box O-1
ATG: Autophagy-related proteins
PGC-1α: Peroxisome proliferator-activated receptor-γ coactivator-1α
NRF-1: Nuclear respiratory factor 1
NRF-2: Nuclear respiratory factor 2
TFAM: Mitochondrial transcription factor A
mtDNA: Mitochondrial DNA
GTPase: Guanosine triphosphatase
OPA1: Optic atrophy-1
MFN1: Mitofusin-1
DRP1: Dynamin-related protein 1
PINK1: PTEN-induced putative kinase-1
GSH: Glutathione
GSSG: Oxidized glutathione
HT: Hydroxytyrosol
